# Impact of Microplastic Exposure on Blood Glucose Levels and Gut Microbiota: Differential Effects under Normal or High-Fat Diet Conditions

**DOI:** 10.3390/metabo14090504

**Published:** 2024-09-18

**Authors:** Manjin Xu, Huixia Niu, Lizhi Wu, Mingluan Xing, Zhe Mo, Zhijian Chen, Xueqing Li, Xiaoming Lou

**Affiliations:** Department of Environmental Health, Zhejiang Provincial Center for Disease Control and Prevention, Hangzhou 310051, China; margexmj@outlook.com (M.X.); niuhuixia1118@163.com (H.N.); lzhwu@cdc.zj.cn (L.W.); mlxing@cdc.zj.cn (M.X.); zhmo@cdc.zj.cn (Z.M.); zhjchen@cdc.zj.cn (Z.C.)

**Keywords:** microplastics, polystyrene, high-fat diet, blood glucose, gut microbiota

## Abstract

Microplastics are emerging pollutants that have garnered significant attention, with evidence suggesting their association with the pathogenesis of type 2 diabetes mellitus. In order to assess the impact of polystyrene microplastic exposure on alterations in the gut microbiota and the subsequent implications for glucose dysregulation under different dietary conditions in mice, we investigated the effects and disparities in the blood glucose levels induced by polystyrene microplastic exposure in mice fed a high-fat diet versus those fed a normal diet. Using 16S rRNA sequencing and bioinformatics analyses, we explored the dynamic changes and discrepancies in the gut microbiota stability induced by polystyrene microplastic exposure under varied dietary conditions, and we screened for gut genera associated with the potential of polystyrene microplastics to disrupt glucose homeostasis. Our findings indicate that a high-fat diet resulted in abnormal mouse body weight, energy intake, blood glucose levels and related metabolic parameters. Additionally, polystyrene microplastic exposure exacerbated the glucose metabolism disorders induced by a high-fat diet. Furthermore, the composition and diversity of the mouse gut microbiota were significantly altered following microplastic exposure, with 11 gut genera exhibiting a differential presence between mice fed a high-fat diet combined with microplastic exposure compared to those fed a normal diet with microplastic exposure. Moreover, *Ucg-009* played an intermediary role in the association between a high-fat diet and the fasting blood glucose. Hence, our study demonstrates that polystyrene microplastic exposure exacerbates high-fat diet-induced glucose metabolism disorders, whereas its impact on the blood glucose under normal dietary conditions is not significant, highlighting the differential influence attributable to distinct alterations in characteristic gut genera.

## 1. Introduction

Microplastics (MPs) are plastic fragments and particles with diameters smaller than 5 mm, which are formed by physical and chemical processes [1]. They have become a global environmental pollutant, and in recent years, microplastics have been further categorized into micro-sized plastics (MPs) and nano-sized plastics (NPs) [2]. Despite being conceptualized only two decades ago, extensive scientific attention is now focused on their distribution, migration, and transformation in the environment, as well as their impact on human health. As a novel pollutant, the potential threats posed by microplastics to ecological environments and human health warrant systematic investigation. Humans may ingest up to 5 g of microplastics per week, and due to their small size, these particles can circulate through the bloodstream, accumulating in various tissues and organs, including the liver, kidneys, spleen, lungs, and placenta, potentially exerting localized cellular and tissue toxicity [3,4]. Long-term exposure to microplastics may disrupt normal immune system function [5], induce chronic inflammatory responses [6], and cause cellular damage [7]. Existing research indicates that microplastic exposure may increase the risk of chronic diseases such as cardiovascular diseases and diabetes [8,9]. The exposure pathways include ingestion via food, intake through drinking water, and inhalation through air contact. Among these routes, ingestion via the digestive system is one of the primary ways microplastics enter organisms, where they can colonize, particularly in the gastrointestinal tract, affecting physiological processes [10]. Microplastics have been detected in human feces, confirming their entry into the human gut and emphasizing the potential impact of microplastics on health through the gut microbiota [11].

Diabetes mellitus is a chronic disease characterized by high blood sugar levels, directly or indirectly caused by insulin deficiency. Globally, the number and proportion of diabetes patients continue to rise. According to the International Diabetes Federation (IDF), as of 2021, there were approximately 537 million diabetes patients worldwide, with China alone having 141 million, equating to a prevalence of 12.8%, predominantly type 2 diabetes [12]. Diabetes has been identified by the United Nations and the World Health Organization as one of the five priority non-communicable diseases to address in global health action plans. The increasing prevalence of diabetes and the substantial rise in healthcare costs place significant burdens on societies, economies, and healthcare systems worldwide [13]. A high-fat diet is recognized as a risk factor contributing to the onset and exacerbation of diabetes, influencing blood glucose metabolism levels. Therefore, it is crucial to deepen understanding of the development of diabetes induced by a high-fat diet and propose new treatment and prevention strategies for comprehensive disease management.

There is substantial evidence suggesting a close association between microplastic exposure and the onset and progression of diabetes. Wang et al. found that mice exhibited impaired glucose tolerance and hepatic lipid deposition following high−dose microplastic administration [14]. Insulin resistance in mice induced by microplastic exposure may also be related to metabolic reprogramming of the gut-liver axis [9]. Importantly, numerous studies indicate that disruptions to glucose metabolism caused by microplastic exposure are linked to alterations in the gut microbiota. We hypothesize that microplastics may impact glucose metabolism through changes in the gut microbiota, exacerbated by a high-fat diet. In this study, we established a mouse model of a high-fat diet combined with polystyrene microplastic exposure, measured relevant indicators of blood glucose metabolism, and performed 16S rRNA sequencing and bioinformatics analysis on collected fecal samples at the phylum and genus levels. Subsequently, we conducted statistical analyses to identify gut genera associated with the role of polystyrene microplastics in glucose dysregulation under different dietary patterns. Finally, we analyzed the mediating effects of relevant gut genera. This research will contribute to a comprehensive understanding of the impact of the gut microbiota on glucose dysregulation induced by microplastic exposure under different dietary patterns, providing insights into the toxicological effects of microplastics and raising concerns about their harm to high-risk populations exposed to high-fat diets.

## 2. Materials and Methods

### 2.1. Chemicals

The 5 μm monodisperse polystyrene microspheres used in this experiment were purchased from the Tianjin BaseLine ChromTech Research Center (Tianjin, China). The samples were prepared as a 2.5% *w*/*v* milky solution, with an initial water suspension concentration of 250 mg/10 mL. The microplastic solution was diluted to 16 mg/mL using deionized water and stored at 4 °C. Particle characterization of the microplastics used in the experiment was conducted using a Kurt particle size analyzer. As shown in Figure S1, the polystyrene microspheres exhibited good mass quality, with no significant differences in the particle size, all averaging around 5 μm.

### 2.2. Experimental Animals and Exposure Protocol

**Figure 1:**
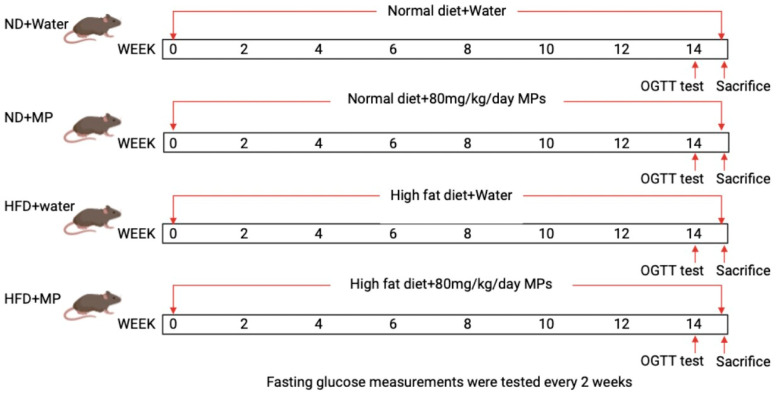
Experimental design.

Prior to commencement, the study protocol was approved by the Zhejiang Chinese Medical University Laboratory Animal Research Center (No. 20230213-07), ensuring compliance with ethical standards in animal research. SPF−grade male C57BL/6 mice, 4–6 weeks old, with uniform genetic backgrounds, were obtained from SLAC Laboratory Animal Co., Ltd. (Shanghai, China). The mice were fed diets from Hangzhou Hangsi Biotechnology Co., Ltd. (Hangzhou, China): a 10 kcal% normal diet and a 60 kcal% high-fat diet, with energy densities of 3.85 kcal/g and 5.24 kcal/g, respectively. The experimental design is shown in Figure 1. After acclimatization for seven days in an animal facility with the temperature maintained at 22 °C, humidity at 55 ± 15%, and a 12 h light-dark cycle, 48 mice were randomly divided into four groups (n = 12/group): normal diet control (ND + water), normal diet microplastic (ND + MP), high-fat diet control (HFD + water), and high-fat diet microplastic (HFD + MP). During the experimental period, mice in the ND + MP and HFD + MP groups were orally gavaged daily with 80 mg/kg body weight of microplastic suspension, following dosage references from previous studies [15]. Mice in the ND + water and HFD + water groups were administered an equivalent volume of high-purity water by oral gavage. The experiment lasted for 14 weeks, during which biweekly measurements were taken for the body weight, food intake, water consumption, and fasting blood glucose. Prior to euthanasia at week 14, fecal samples were collected from each mouse, immediately frozen in liquid nitrogen, and stored at −80 °C. After a 12-h fast, final measurements of the body weight and fasting blood glucose were taken, blood was collected via retro−orbital bleeding, and the mice were euthanized via cervical dislocation. Tissues, including from the pancreas, colon, cecum, small intestine, liver, and epididymal fat pads, were collected and weighed. The experimental procedures were conducted at the Zhejiang Chinese Medical University Laboratory Animal Research Center.

### 2.3. Oral Glucose Tolerance Test

At week 14 of the experiment, after a 12−hour fast, the mice were orally administered a glucose solution at a dose of 2 g/kg body weight. Blood samples were collected via tail vein puncture at 0, 30, 60, 90, 120, and 150 min post−glucose administration, and the blood glucose levels were measured using a glucometer. GraphPad was used to plot the blood glucose curve and calculate the area under the curve.

### 2.4. 16S rRNA High-Throughput Sequencing

Genomic DNA of the gut microbiota from the fecal samples was extracted using the Mag—bind Soil DNA kit. The DNA purity and concentration were assessed by 1% agarose gel electrophoresis. Barcoded primers were used to amplify the diluted genomic DNA following the standard protocol of the Illumina MiSeq platform using the TruSeq^®^ DNA PCR—Free Sample Preparation Kit. Quantification was performed with QuantiFluor™—ST, and the sequences were subjected to quality filtering and base trimming. The sequencing was performed by Shanghai Applied Protein Technology Co., Ltd. (Shanghai, China).

### 2.5. Gut Microbiota Analysis

The operational taxonomic units (OTUs) were assigned using the Uparse algorithm (version 7.0.1090), with a sequence similarity threshold of 97%. Alpha diversity indices, including Shannon, Simpson, and Chao1, were calculated to assess the community diversity and richness. Principal coordinate analysis (PCoA) based on the weighted and unweighted UniFrac distances was conducted to assess the beta diversity among the study groups. The composition and abundance of the gut microbiota at the phylum and genus levels were analyzed, visualized using bar graphs, and statistically tested for significance using the linear discriminant analysis effect size (LEfSe) and other appropriate statistical methods.

### 2.6. Statistical Analysis

Statistical analyses and graphing of the experimental data were performed using R version 4.3.0, SPSS version 26.0.0.0, and GraphPad Prism version 10.2.1. The results are presented as means ± standard deviations ($\bar{x}$ ± *SD*). A one-way ANOVA was used to test for statistical differences among the four groups, and *t*-tests were employed for comparisons between two groups. Bootstrap methods were used to validate the mediating effects of the gut microbiota. The direct effects of high-fat diet exposure on the fasting blood glucose outcomes and the indirect effects mediated by the gut microbiota were estimated, with the proportion of mediation calculated as the “indirect effect/total effect”. Statistical significance was set at *α* = 0.05.

## 3. Results

### 3.1. Effects of Polystyrene Microplastic Exposure on Mouse Body Weight Gain, Energy Intake, and Organ Weights

**Figure 2:**
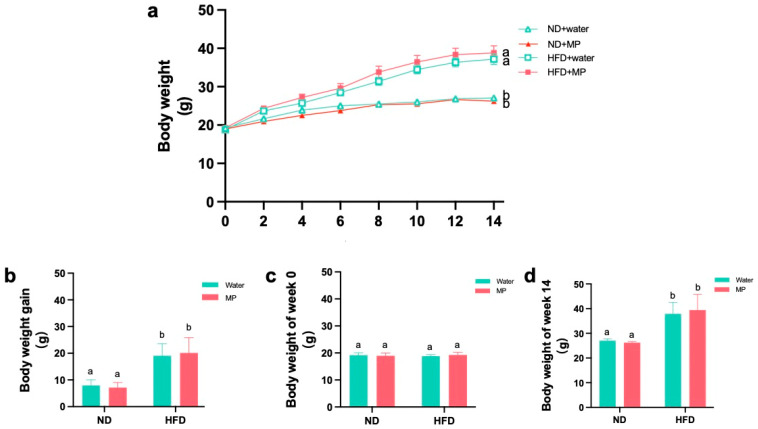
Effects of polystyrene microplastics on the body weight of mice: (**a**) the weight change of the mice at 14 weeks; (**b**) the weight gain of the mice at 14 weeks; (**c**) the weight of the mice at the 0th week; and (**d**) the weight of the mice at the 14th week. Different letters indicate that there is statistical significance in the comparison between groups, *p* < 0.05.

The biweekly changes in the mouse body weight are depicted in Figure 2a. The bar graphs showing the mouse body weights at week 0 and week 14 are presented in Figure 2b and Figure 2c, respectively. The bar graph illustrating the weight gain during the experimental period is shown in Figure 2d. The results indicate that the high-fat diet led to significant weight gain. However, there were no significant differences in body weight between the control and microplastic-exposed mice under both dietary conditions, suggesting that polystyrene microplastic exposure did not significantly affect the mouse body weight.

Details of the average food intake and energy consumption per group are summarized in Tables S1 and S2, respectively. The results show that the high-fat diet led to increased organ weights (*p* < 0.05). However, there were no significant differences in the food intake and energy consumption between the control and microplastic-exposed mice under both dietary conditions, indicating that polystyrene microplastic exposure did not significantly influence the energy intake in the mice.

The absolute and relative weights of various organs and tissues are presented in Supplementary Tables S3 and S4. The absolute weight results indicate that the high-fat diet significantly increased the pancreatic, adipose, and colonic weights in the mice (*p* < 0.05). However, there were no significant differences in the absolute organ weights between the control and microplastic-exposed mice under the different dietary conditions. The relative weight results show that the high-fat diet significantly increased the relative weights of the pancreas and epididymal fat pads, while decreasing the relative weight of the cecal tissue (*p* < 0.05). Again, there were no significant differences in the relative organ weights between the control and microplastic-exposed mice under the different dietary conditions, indicating that microplastic exposure had no significant impact on the organ weights between the dietary groups.

### 3.2. Polystyrene Microplastic Induces Abnormal Fasting Blood Glucose in Mice

**Figure 3:**
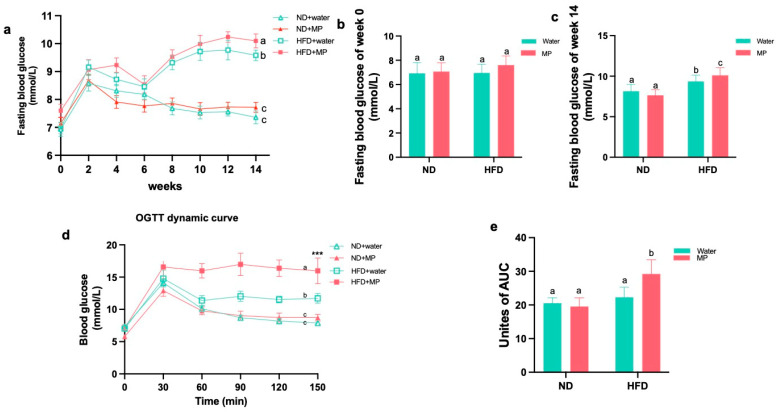
Effects of polystyrene microplastics on the fasting blood glucose and glucose tolerance in mice: (**a**) fasting blood glucose changes of the mice at 14 weeks; (**b**) fasting blood glucose of the mice at 0 week; (**c**) fasting blood glucose of the mice at 14 weeks; (**d**) blood glucose changes of the mice during OGTT test; and (**e**) area under OGTT curve. Different letters indicate that there is statistical significance in the comparison between groups, *p* < 0.05.

The fasting blood glucose levels were measured biweekly throughout the experiment, and the changes were plotted in line and bar graphs, as shown in Figure 3a. Statistical analysis of the data revealed no significant differences in the fasting blood glucose among the groups from week 0 to week 12. However, starting from week 8, significant differences in the fasting blood glucose between the high-fat diet control group and the normal diet control group mice were observed (*p* < 0.05). Additionally, under the normal diet regimen, there were no significant differences in the fasting blood glucose between the microplastic-exposed mice and the control mice. In contrast, under the high-fat diet regimen, microplastic exposure did not significantly affect the fasting blood glucose levels at 12 weeks but resulted in a significant increase at 14 weeks (*p* < 0.05). These findings indicate that while the high-fat diet induced changes in the mouse blood glucose, polystyrene microplastic exposure alone did not affect the fasting blood glucose significantly. However, co-exposure with a high-fat diet exacerbated the effects of the diet on the mouse fasting blood glucose.

### 3.3. Polystyrene Microplastic Induces Abnormal Glucose Tolerance in Mice

A few days before the end of the 14th week of exposure, mice fasted for 12 h were subjected to an OGTT (oral glucose tolerance test). The blood glucose levels were measured and recorded during the test, and the results were statistically analyzed, as shown in Figure 3d. According to the experimental results, after glucose solution administration, the blood glucose levels in the mice rose rapidly, with no significant differences among the groups at 30 min. Subsequently, from 30 min onward, the blood glucose began to decline. The mice fed a normal diet exhibited a faster decline in the blood glucose levels compared to those fed a high-fat diet, and from 60 min onward, significant statistical differences in the blood glucose changes emerged between the high-fat diet control group and the normal diet control group (*p* < 0.05). Under the different dietary conditions, there were no significant differences in glucose tolerance between the microplastic-exposed and control mice under the normal diet. However, under the high-fat diet, the mice exposed to microplastics showed slower declines in the blood glucose levels, with significant statistical differences emerging from 60 min onward (*p* < 0.05). Calculations of the area under the OGTT curve revealed significant differences between the high-fat diet control group and the normal diet control group (*p* < 0.05). In the normal diet model, there were no significant differences in the area under the curve between the microplastic-exposed and control mice. In contrast, in the high-fat diet model, the area under the curve was significantly larger in the microplastic-exposed mice compared to the control mice (*p* < 0.05).

### 3.4. Differences in Gut Microbiota Diversity

**Figure 4:**
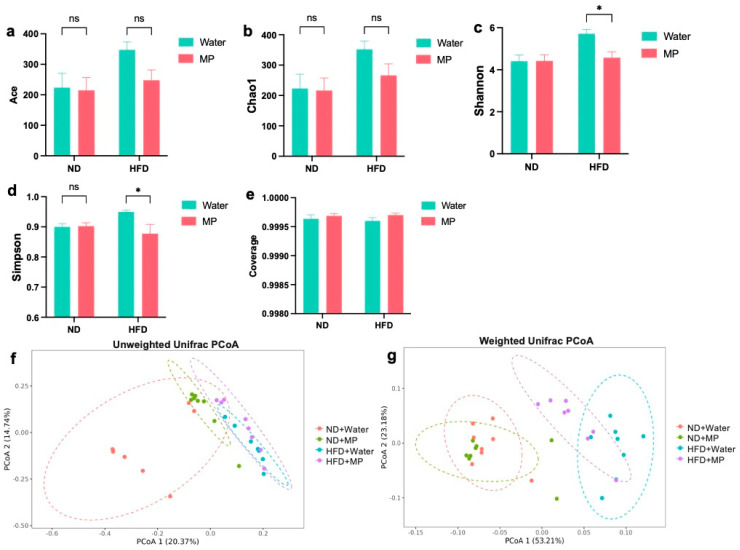
Effects of polystyrene microplastics on the diversity of the intestinal microflora in mice: (**a**) mouse Ace index; (**b**) mouse Chao1 index; (**c**) mouse Shannon index; (**d**) mouse Simpson index; (**e**) mouse Coverage index; (**f**) analysis based on unweighted UniFrac PcoA; and (**g**) analysis based on weighted UniFrac PcoA; * *p* < 0.05.

The Ace and Chao1 indices estimated that under the normal diet regimen, there were no significant differences in the Ace and Chao1 indices between the microplastic-exposed mice and the control mice. Under the high-fat diet regimen, the microplastic-exposed mice showed slightly lower Ace and Chao1 indices compared to the control mice, but the differences were not statistically significant. Overall, these results indicate that polystyrene microplastics had no significant impact on the species diversity of the gut microbiota. The results of the Shannon and Simpson indices are shown in Figure 4c and Figure 4d, respectively. In the normal diet regimen, there were no statistically significant differences in the Shannon and Simpson indices between the microplastic−exposed mice and the control mice. However, under the high-fat diet regimen, the microplastic−exposed mice exhibited significantly lower Shannon and Simpson indices compared to the control mice (*p* < 0.05), indicating a significant effect. Taken together, these results suggest that polystyrene microplastic exposure affects the richness and evenness of the gut microbiota in mice fed a high-fat diet. As shown in Figure 4e, all the groups had a Coverage index >0.9995, indicating high sample authenticity and representativeness. These sequencing results demonstrate that polystyrene microplastic exposure alters the α-diversity of the gut microbiota in mice under the high-fat diet regimen, with distinct differences compared to the normal diet regimen.

The β-diversity assessed by unweighted UniFrac is depicted in Figure 4f. The results show significant differences at the OTU level between the mice fed a high-fat diet and those fed a normal diet. In both dietary models, there were differences in the OTU levels between the microplastic−exposed and control groups. The weighted UniFrac results in Figure 4g indicate significant differences in the OTU levels between the mice fed a high-fat diet and those fed a normal diet. Moreover, under both dietary models, there were differences in the OTU levels between the microplastic-exposed and control groups, with a more pronounced difference in the high-fat diet model, characterized by a reduced overlap. These results demonstrate that polystyrene microplastic exposure induces changes in the β-diversity of the gut microbiota in mice, with more significant changes observed under the high-fat diet compared to the normal diet.

### 3.5. Identification of Characteristic Gut Microbiota Differences

**Figure 5:**
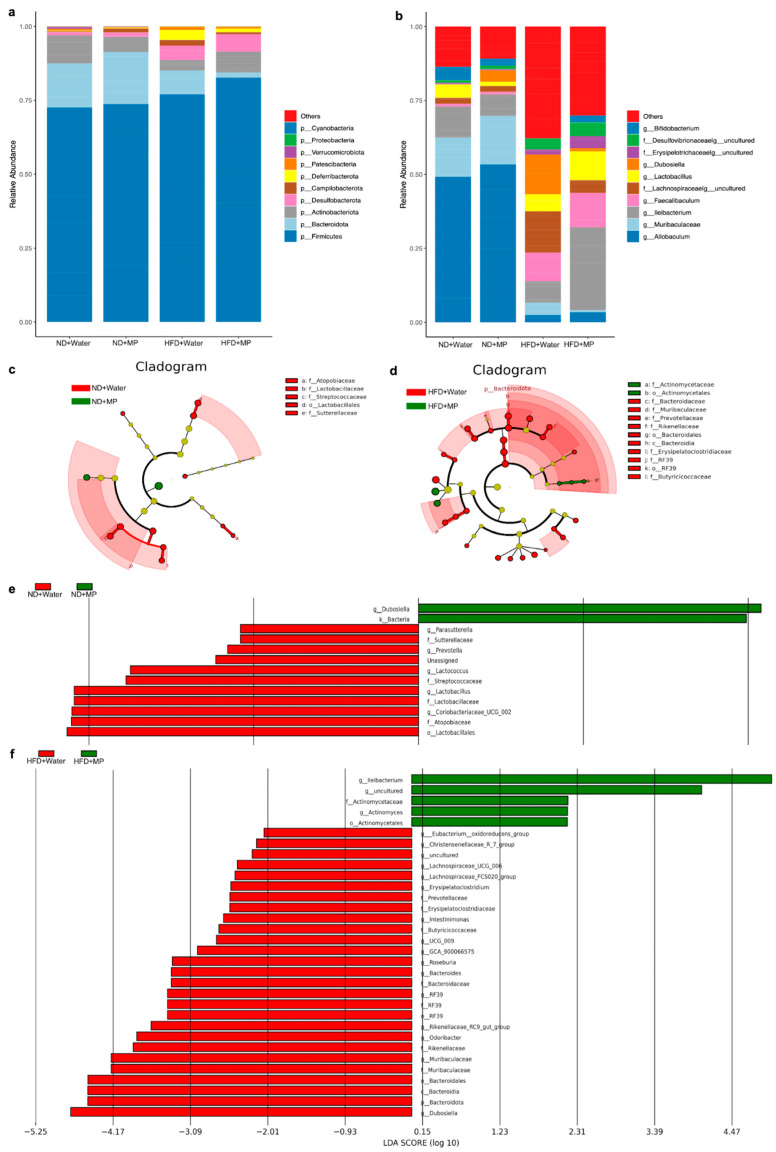
Effects of polystyrene microplastic exposure on the gut microbiota composition in mice: (**a**) changes in the gut microbiota composition at the phyla level; (**b**) changes in the gut microbiota composition at the genera level; (**c**,**e**) cladogram analysis map and bar chart of the LDA score (log10) distribution of the gut microbiota in the normal diet control group and microplastic group, where red represents microbial communities that play an important role in the (ND + Water) group, green represents microbial communities that play an important role in the (ND + MP) group, yellow represents microbial communities that did not play an important role in either group; and (**d**,**f**) cladogram analysis map and bar chart of the LDA score (log10) distribution of the gut microbiota in the high-fat diet control group and microplastic group, where red represents microbial communities that play an important role in the (HFD + Water) group, green represents microbial communities that play an important role in the (HFD + MP) group, and yellow represents microbial communities that did not play an important role in either group. The current LDA threshold score is over 2.

Based on the 16S rRNA sequencing results, the gut microbiota composition and relative abundance at the phylum level were analyzed, as shown in Figure 5a. The results indicate that *Firmicutes*, *Bacteroidota*, *Actinobacteriota*, *Desulfobacterota*, *Campilobacterota*, and *Deferribacterota* were the most abundant phyla in the mouse gut microbiota, with *Firmicutes* and *Bacteroidota* being predominant. Comparing the different treatment groups, significant changes were observed in the gut microbiota at the phylum level in the mice exposed to polystyrene microplastics. Under the normal diet regimen, the microplastic-exposed mice showed increased relative abundance of *Firmicutes*, *Bacteroidota*, *Desulfobacterota*, *Campilobacterota*, and *Deferribacterota* compared to the control mice, while *Actinobacteriota* exhibited a decreasing trend. Under the high-fat diet regimen, the microplastic-exposed mice exhibited increased relative abundance of *Firmicutes*, *Actinobacteriota*, and *Desulfobacterota*, and decreased relative abundance of *Bacteroidota*, *Campilobacterota*, and *Deferribacterota*, compared to the control mice.

Further analysis at the genus level was conducted to explore the impact of polystyrene microplastic exposure on the gut microbiota’s relative abundance, as shown in Figure 5b. The results reveal that the mouse gut bacteria at the genus level include *Allobaculum*, *Muribaculaceae*, *Ileibacterium*, *Faecalibaculum*, *Lachnospiraceae*, *Lactobacillus,* and *Dubosiella*, among others. Under the normal diet regimen, the microplastic−exposed mice showed increased relative abundance of *Ileibacterium* and *Lactobacillus*, while *Allobaculum*, *Muribaculaceae*, *Faecalibaculum*, *Lachnospiraceae*, and *Dubosiella* exhibited decreased relative abundance compared to the control mice. Under the high-fat diet regimen, the microplastic-exposed mice exhibited increased relative abundance of *Allobaculum*, *Muribaculaceae*, *Ileibacterium*, *Faecalibaculum*, and *Lactobacillus*, while *Lachnospiraceae* and *Dubosiella* exhibited decreased relative abundance compared to the control mice.

The LEfSe analysis further compared the significant differences in the gut microbiota composition between the microplastic-exposed and control groups, identifying characteristic gut microbiota. The LEfSe evolutionary branch diagram and LDA value distribution histogram for the intergroup comparisons are shown in Figure 5c–f. The results indicate that under the normal diet regimen, the microplastic−exposed mice exhibited increased relative abundance of *g__Dubosiella* and *k__Bacteria* compared to the control mice. Additionally, under the high-fat diet regimen, the microplastic−exposed mice showed significantly increased relative abundance of *g__Ileibacterium*, *g__uncultured*, *g__Actinomycetales*, *g__Actinomyces*, and *g__Actinomycetales* compared to the control mice. These results underscore the significant changes in the gut microbiota composition following polystyrene microplastic exposure in mice.

### 3.6. Changes in Gut Microbiota Genera upon Microplastic Exposure under Different Dietary Models

**Figure 6:**
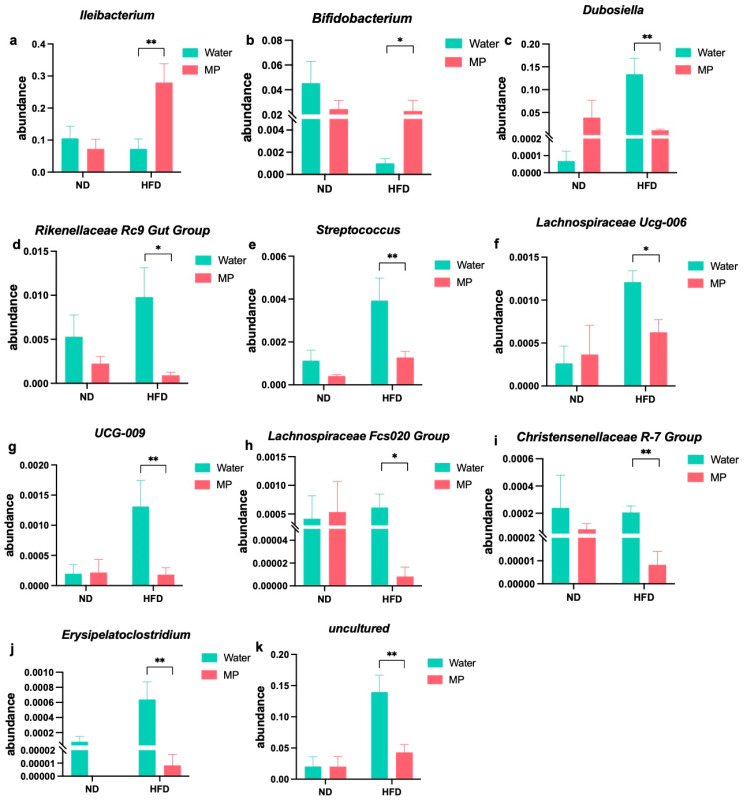
The difference in the intestinal bacteria at different levels caused by exposure to polystyrene microplastics in mice fed with different dietary patterns: (**a**) the change in relative abundance of *Ileibacterium*; (**b**) the change in relative abundance of *Bifidobacterium*; (**c**) the change in relative abundance of *Dubosiella*; (**d**) the change in relative abundance of *Rikenellaceae_RC9_gut_group*; (**e**) the change in relative abundance of *Streptococcus*; (**f**) the relative abundance of *Lachnospiraceae_UCG-006*; (**g**) the change in relative abundance of *UCG-009*; (**h**) change in relative abundance of *Lachnospiraceae Fcs020 Group*; (**i**) the change in relative abundance of *Christensenellaceae_R-7_group*; (**j**) the change in relative abundance of *Erysipelatoclostridium*; and (**k**) the change in relative abundance of *uncultured*. * *p* < 0.05, ** *p* < 0.01.

At the genus level, differential analysis of the gut microbiota was conducted to explore the impact of polystyrene microplastic exposure under different dietary models, as illustrated in Figure 6. The results indicate significant changes in the relative abundance of the gut microbiota genera between the microplastic-exposed and control groups under both the normal diet and high-fat diet regimens. Under the normal diet regimen, the microplastic-exposed mice showed significant changes in relative abundance of seven genera compared to the control mice. In contrast, when comparing the microplastic-exposed mice under the high-fat diet regimen to the control mice, 12 genera exhibited significant changes. Specifically, 11 genera showed significant differences (*p* < 0.05) following microplastic exposure under the high-fat diet regimen compared to the control group. These genera include *Ileibacterium*, *Bifidobacterium*, *Dubosiella*, *Rikenellaceae Rc9 Gut Group*, *Streptococcus*, *Lachnospiraceae Ucg-006*, *Ucg-009*, *Lachnospiraceae Fcs020 Group*, *Christensenellaceae R-7 Group*, *Erysipelatoclostridium*, and one unclassified genus. Among these, the first two genera exhibited significantly increased relative abundance following microplastic exposure under the high-fat diet regimen, while the latter nine genera showed significantly decreased relative abundance. These findings underscore the differential impact of polystyrene microplastic exposure on the relative abundance of the gut microbiota genera depending on the dietary context, highlighting notable shifts, particularly under conditions of high-fat diet consumption.

### 3.7. Gut Microbiota-Mediated Indirect Effects

The above results suggest that polystyrene microplastic exposure alone does not affect glucose tolerance in mice. However, when exposed simultaneously with a high-fat diet, microplastics exacerbate the diet’s impact on glucose tolerance in mice. The gut microbiota may play a key role in the different effects of microplastic exposure under different dietary conditions. To further explore the role of differential changes in specific genera in relation to the outcome of the fasting blood glucose, we conducted mediation analysis to assess the specific gut microbiota−mediated indirect effects and proportions.

Among the 11 genera showing differential changes, we found that *Ucg-009* exhibited a significant mediation effect in the association between a high-fat diet and the fasting blood glucose outcome. The results of the mediating analysis are shown in Table 1. The proportion mediated by *Ucg-009* was 0.6308 (*p* < 0.05).

## 4. Discussion

As a new environmental contaminant, microplastics have garnered widespread attention. Increasing evidence shows that microplastics can be ingested orally and exert toxic effects on various target organs, including inducing inflammation, neurotoxicity, and oxidative stress, and impairing immune and circulatory systems. However, due to their relatively low toxicity or less pronounced effects, their harmfulness is often overlooked. A high-fat diet has been widely recognized as a risk factor for diabetes [16]. Previous studies found that oral administration of fPS MP to high-fat diet-induced obese mice exacerbates impaired glucose metabolism and insulin resistance, and it promotes systemic inflammation [17]. Leaky gut syndrome (LGS) may be caused by HFD, leading to MP deposition in the intestinal mucosa, causing inflammation of the inner layer of the intestinal mucosa, exacerbating insulin resistance and affecting insulin secretion, thereby affecting the blood glucose levels [18]. Yet, research on the toxic effects and mechanisms of microplastic exposure under high-fat diet conditions remains limited. In our study, based on observations of metabolic markers related to blood glucose and changes in the gut microbiota under different dietary conditions, we explored the association between microplastic exposure and blood glucose impacts in mice.

We designed four exposure groups: normal diet control (ND + water), normal diet microplastic (ND + MP), high-fat diet control (HFD + water), and high-fat diet microplastic (HFD + MP). By comparing the phenotypic differences among these groups, we found that microplastic exposure exacerbated the blood glucose metabolism disruption in the mice fed a high-fat diet while showing no significant impact on the blood glucose in the mice fed a normal diet. Okamura et al. also evaluated the gut outcomes associated with a high-fat diet and MP exposure, and some of the conclusions are similar to our study. They found that a microplastic-induced disruption to blood glucose metabolism only occurred in mice fed a high-fat diet [18]. However, Okamura et al.’s article focuses on the endocrine and metabolic systems, with a particular emphasis on evaluating the gut pathophysiology, while our article focuses on differential changes in the gut microbiota and conducts mediation analysis of these differential changes, with a focus on statistical methods. Comparing the research results, although *Firmicutes* and *Bacteroideta* were both the phyla with the highest content in both studies, our results showed that *Firmicutes* had the highest relative abundance, while Okamura et al.’s study exhibited a higher relative abundance of *Bacteroideta*. *Firmicutes* and *Bacteroidetes* are known as the main bacteria in the gut microbiota, and their relative abundance changes are related to disease, health, obesity, and dietary habits. This difference may be explained by differences in the dietary energy design (3.85 kcal/g and 5.24 kcal/g in our study, and 345 kcal/100 g and 459 kcal/100 g in Okamura et al.’s study, respectively), or methodological differences in the sample processing and DNA analysis [19]. Moreover, in Okamura et al.’s study, microplastics were provided ad libitum. Our study used the method of gavage administration for microplastic exposure, and the concentrations of the microplastics in the two studies were also different. These differences in experimental conditions may lead to different bacterial abundances. Although there are differences in the abundance of the gut microbiota, combining the results of the two studies, we hypothesize that microplastic exposure aggravates the blood glucose metabolism disruption under a high-fat diet, and the gut microbiota may play a role in this process.

At the genus level, our study identified differential changes in 11 genera, such as *Ileibacterium*, *Bifidobacterium*, and *Dubosiella*, following microplastic exposure under different dietary conditions. Among these, *Ucg-009* was found to mediate the association between a high-fat diet and the fasting blood glucose outcome. The gut microbiota plays critical roles in regulating various pathways in human metabolism and physiology, including immune, energy, lipid, and glucose metabolism [20]. Dysbiosis of the gut microbiota has been implicated in the pathogenesis of obesity and type 2 diabetes [21]. *Ileibacterium*, considered a harmful bacterium, has been positively correlated with blood lipid levels and metabolic disorders [22]. It has also been implicated in type 2 diabetes and diabetic nephropathy [23]. Additionally, Xiao et al. demonstrated that decreased abundance of *Ileibacterium* and *Bifidobacterium* positively regulated the gut microbiota and suppressed blood glucose disorders [24]. Similarly, our experimental results showed significant changes in *Ileibacterium* under different dietary feeding patterns with microplastic exposure, where its relative abundance significantly increased under a high-fat diet but showed no significant change under a normal diet with microplastic exposure. Microplastic exposure may aggravate the blood glucose disruption induced by a high-fat diet by increasing the levels of *Ileibacterium*.

*Dubosiella* has been linked to the development of certain diseases, such as diabetes [25], and is widely used as a probiotic in the medical field [26]. *Christensenellaceae R-7 Group* is considered beneficial in regulating diabetes-related gut types [27]. In our study, we found significant reductions in *Dubosiella* and *Christensenellaceae R-7 Group* following microplastic exposure under a high-fat diet, which differed from the changes observed under a normal diet with exposure to microplastics. *Rikenellaceae RC9 Gut Group* has been reported to promote the expression of fat synthesis genes and increase obesity [28]. Keisiii et al. found that the *Lachnospiraceae* family is associated with metabolic dysfunction and the development of obesity and diabetes in mice [29]. However, in our experimental results, the relative abundances of *Rikenellaceae RC Gut Group* and various genera within *Lachnospiraceae* were decreased under microplastic exposure in a high-fat diet model. Further research is needed to determine the role of *Rikenellaceae RC Gut Group* and the *Lachnospiraceae* family in exacerbating the blood glucose disruption induced by microplastics. It is reported that *Ucg-009* is negatively correlated with HOMA-IR, HDL-C, ALT, AST, TC, and lipopolysaccharide (LPS), indicating that *Ucg-009* is beneficial to hypoglycemia and hypolipidemia in diabetic mice [30]. However, we found that *Ucg-009* significantly decreased after exposure to microplastics under high-fat diet feeding, and this change in the relative abundance was validated to play a mediating role in the association between high-fat diet microplastic exposure and blood glucose changes. This result indicated that exposure to high-fat dietary microplastics has adverse effects on blood glucose by reducing *Ucg-009*. Thus, the differential effects of microplastics on blood glucose in mice under different dietary conditions could be attributed to differential changes in characteristic gut genera. 

To the best of our knowledge, there was a limited understanding of the exacerbation of glucose metabolism disorders under high-fat diet conditions due to microplastic exposure and the role of the gut microbiota therein. Our findings reveal significant changes in the blood glucose and distinct gut microbiota in mice exposed to microplastics under a high-fat diet pattern. Statistical methods were applied to validate the mediating role of the gut microbiota, providing evidence for further exploration of characteristic gut microbiota in the context of microplastic-induced glucose changes under high-fat diet conditions. This study offers new insights into the toxicological effects and health risks associated with microplastic exposure. Further mechanistic studies and clinical trials are necessary to comprehensively explore the roles and specific mechanisms of microplastics in blood glucose metabolism in high-fat diet populations, which could provide valuable insights for preventing and treating diabetes in high-risk populations.

## 5. Conclusions

This study investigated the differential effects of microplastic exposure on the blood glucose levels and gut microbiota homeostasis in mice fed normal and high-fat diets. The results demonstrate that microplastic exposure exacerbates the blood glucose disruption in mice fed a high-fat diet while showing no significant impact on those fed a normal diet. Additionally, the differential effects of microplastics on the blood glucose in mice under different dietary conditions may be associated with differential changes in characteristic gut genera. This study contributes to a better understanding of the health risks posed by microplastics and their potential impact on exacerbating glucose metabolic disruption in populations with a high-fat diet.

## Figures and Tables

**Table 1:** Analysis of the mediating effect of *Ucg-009*.

	Effect Size	Bootstrap 95% CI		*p*	Proportion Mediated
		Lower	Upper		
Direct effect	0.5361	0.0353	1.2	0.026	0.3692
Indirect effect	0.3139	−0.465	1.08	0.444	0.6308
Total effect	0.85	0.0632	1.68	0.024	1

## Data Availability

The original contributions presented in the study are included in the article/Supplementary Mmaterial, further inquiries can be directed to the corresponding authors.

## Supplementary Materials

The following supporting information can be downloaded at https://www.mdpi.com/article/10.3390/metabo14090504/s1, Figure S1: Kurt particle size analysis of polystyrene microplastic microspheres (data provided by Tianjin Bessler chromatographic Technology Development Center); Table S1: Average food intake of the mice; Table S2. Average energy intake of the mice; Table S3. Absolute weight of the mouse viscera and tissue; Table S4. Relative weight of the organs and tissues in the mice.

## References

[1] Thompson R., Olsen Y., Mitchell R., Davis A., Rowland S., John A., McGonigle D., Russell A. (2004). Lost at Sea: Where Is All the Plastic?. Science.

[2] Ter Halle A., Jeanneau L., Martignac M., Jardé E., Pedrono B., Brach L., Gigault J. (2017). Nanoplastic in the North Atlantic Subtropical Gyre. Environ. Sci. Technol..

[3] Wright S., Kelly F. (2017). Plastic and Human Health: A Micro Issue?. Environ. Sci. Technol..

[4] Jeong C., Won E., Kang H., Lee M., Hwang D., Hwang U., Zhou B., Souissi S., Lee S., Lee J. (2016). Microplastic Size-Dependent Toxicity, Oxidative Stress Induction, and p-JNK and p-P38 Activation in the Monogonont Rotifer (Brachionus Koreanus). Environ. Sci. Technol..

[5] Tang Y., Rong J., Guan X., Zha S., Shi W., Han Y., Du X., Wu F., Huang W., Liu G. (2020). Immunotoxicity of Microplastics and Two Persistent Organic Pollutants Alone or in Combination to a Bivalve Species. Environ. Pollut..

[6] Li B., Ding Y., Cheng X., Sheng D., Xu Z., Rong Q., Wu Y., Zhao H., Ji X., Zhang Y. (2020). Polyethylene Microplastics Affect the Distribution of Gut Microbiota and Inflammation Development in Mice. Chemosphere.

[7] Liu T., Hou B., Wang Z., Yang Y. (2022). Polystyrene Microplastics Induce Mitochondrial Damage in Mouse GC-2 Cells. Ecotoxicol. Environ. Saf..

[8] Marfella R., Prattichizzo F., Sardu C., Fulgenzi G., Graciotti L., Spadoni T., D’Onofrio N., Scisciola L., La Grotta R., Frigé C. (2024). Microplastics and Nanoplastics in Atheromas and Cardiovascular Events. N. Engl. J. Med..

[9] Shi C., Han X., Guo W., Wu Q., Yang X., Wang Y., Tang G., Wang S., Wang Z., Liu Y. (2022). Disturbed Gut−Liver Axis Indicating Oral Exposure to Polystyrene Microplastic Potentially Increases the Risk of Insulin Resistance. Environ. Int..

[10] Li W., Chen X., Li M., Cai Z., Gong H., Yan M. (2022). Microplastics as an Aquatic Pollutant Affect Gut Microbiota within Aquatic Animals. J. Hazard. Mater..

[11] Liebmann B., Köppel S., Königshofer P., Bucsics T., Reiberger T., Schwabl P. (2018). Assessment of Microplastic Concentrations in Human Stool Final Results of a Prospective Study. United Eur. Gastroenterol..

[12] Wang Y., Wei Z., Xu K., Wang X., Gao X., Han Q., Wang S., Chen M. (2023). The Effect and a Mechanistic Evaluation of Polystyrene Nanoplastics on a Mouse Model of Type 2 Diabetes. Food Chem. Toxicol..

[13] Perry R., Samuel V., Petersen K., Shulman G. (2014). The Role of Hepatic Lipids in Hepatic Insulin Resistance and Type 2 Diabetes. Nature.

[14] Wang Q., Wu Y., Zhang W., Shen T., Li H., Wu J., Zhang L., Qin L., Chen R., Gu W. (2022). Lipidomics and Transcriptomics Insight into Impacts of Microplastics Exposure on Hepatic Lipid Metabolism in Mice. Chemosphere.

[15] Huang D., Zhang Y., Long J., Yang X., Bao L., Yang Z., Wu B., Si R., Zhao W., Peng C. (2022). Polystyrene Microplastic Exposure Induces Insulin Resistance in Mice via Dysbacteriosis and Pro-Inflammation. Sci. Total Environ..

[16] Assmann G., Sacks F., Awad A., Ascherio A., Bonanome A., Berra B., Booyse F., Carmena R., Dahlan W., DeLorgeril M. (2002). Dietary Fat Consensus Statements. Am. J. Med..

[17] Lee A.G., Kang S., Yoon H.J., Im S., Oh S.J., Pak Y.K. (2023). Polystyrene Microplastics Exacerbate Systemic Inflammation in High-Fat Diet−Induced Obesity. Int. J. Mol. Sci..

[18] Okamura T., Hamaguchi M., Hasegawa Y., Hashimoto Y., Majima S., Senmaru T., Ushigome E., Nakanishi N., Asano M., Yamazaki M. (2023). Oral Exposure to Polystyrene Microplastics of Mice on a Normal or High-Fat Diet and Intestinal and Metabolic Outcomes. Environ. Health Perspect..

[19] Magne F., Gotteland M., Gauthier L., Zazueta A., Pesoa S., Navarrete P., Balamurugan R. (2020). The Firmicutes/Bacteroidetes Ratio: A Relevant Marker of Gut Dysbiosis in Obese Patients?. Nutrients.

[20] de Vos W., Tilg H., Van Hul M., Cani P. (2022). Gut Microbiome and Health: Mechanistic Insights. Gut.

[21] Turnbaugh P., Ley R., Mahowald M., Magrini V., Mardis E., Gordon J. (2006). An Obesity-Associated Gut Microbiome with Increased Capacity for Energy Harvest. Nature.

[22] Xia F., Xiang S., Chen Z., Song L., Li Y., Liao Z., Ge B., Zhou B. (2021). The Probiotic Effects of AB23A on High-Fat-Diet-Induced Non-Alcoholic Fatty Liver Disease in Mice May Be Associated with Suppressing the Serum Levels of Lipopolysaccharides and Branched-Chain Amino Acids. Arch. Biochem. Biophys..

[23] Wu C., Fei J., Xu Q., Tao Y., Zhou Z., Wang Y., Wu J., Gu H.F. (2022). Interaction between Plasma Metabolomics and Intestinal Microbiome in Db/Db Mouse, an Animal Model for Study of Type 2 Diabetes and Diabetic Kidney Disease. Metabolites.

[24] Xiao N., Ruan S., Mo Q., Zhao M., Feng F. (2023). The Effect of Sodium Benzoate on Host Health: Insight into Physiological Indexes and Gut Microbiota. Foods.

[25] Ai X., Wu C., Yin T., Zhur O., Liu C., Yan X., Yi C., Liu D., Xiao L., Li W. (2021). Antidiabetic Function of Lactobacillus Fermentum MF423-Fermented Rice Bran and Its Effect on Gut Microbiota Structure in Type 2 Diabetic Mice. Front. Microbiol..

[26] Liu T., Chen M., Tu W., Liang Q., Tao W., Jin Z., Xiao Y., Chen L. (2021). Network and 16S rRNA Sequencing-Combined Approach Provides Insightal Evidence of Vitamin K2 for Salt-Sensitive Hypertension. Front. Nutr..

[27] Hu R., Zeng F., Wu L., Wan X., Chen Y., Zhang J., Liu B. (2019). Fermented Carrot Juice Attenuates Type 2 Diabetes by Mediating Gut Microbiota in Rats. Food Funct..

[28] Kang Y., Li Y., Du Y., Guo L., Chen M., Huang X., Yang F., Hong J., Kong X. (2019). Konjaku Flour Reduces Obesity in Mice by Modulating the Composition of the Gut Microbiota. Int. J. Obes..

[29] Kameyama K., Itoii K. (2014). Intestinal Colonization by a Lachnospiraceae Bacterium Contributes to the Development of Diabetes in Obese Mice. Microbes Environ..

[30] Ma Q., Zhai R., Xie X., Chen T., Zhang Z., Liu H., Nie C., Yuan X., Tu A., Tian B. (2022). Hypoglycemic Effects of Lycium Barbarum Polysaccharide in Type 2 Diabetes Mellitus Mice via Modulating Gut Microbiota. Front. Nutr..

