# Supplementary material for: Impact of Microplastic Exposure on Blood Glucose Levels and Gut Microbiota: Differential Effects under Normal or High-Fat Diet Conditions
Source: Metabolites. 2024 Sep 18;14(9):504. doi: 10.3390/metabo14090504 (PMC11433756; doi:10.3390/metabo14090504)
Supplement: Supplementary file 1 [file metabolites-14-00504-s001.zip › metabolites-3169956-supplementary.pdf]

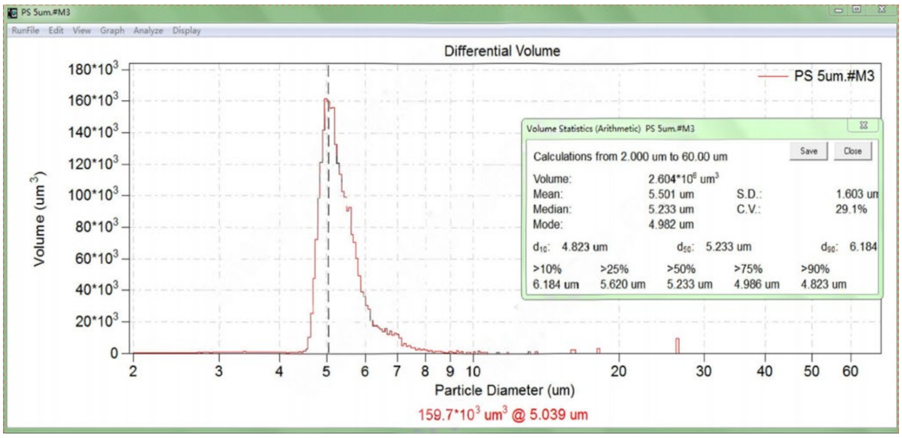

Figure S1. Kurt particle size analysis of polystyrene microplastic microspheres. (data provided by Tianjin Bessler chromatographic Technology Development Center)

Table S1. Average food intake of mice

Weeks	Food intakes（g/d）			
	ND + water	ND + MP	HFD + water	HFD + MP
1-2	2.54 ± 0.09 [a]	2.51 ± 0.08 [a]	7.17 ± 1.31 [b]	8.04 ± 0.34 [b]
3-4	2.31 ± 0.10 [a]	2.13 ± 0.13 [a]	5.15 ± 0.44 [b]	4.71 ± 0.49 [b]
5-6	2.46 ± 0.13 [a]	2.29 ± 0.12 [a]	4.94 ± 0.37 [b]	4.41 ± 0.32 [b]
7-8	2.94 ± 0.13 [a]	2.94 ± 0.15 [a]	5.68 ± 0.20 [b]	5.40 ± 0.49 [b]
9-10	2.30 ± 0.10 [a]	2.18 ± 0.10 [a]	4.70 ± 0.54 [b]	4.10 ± 0.40 [b]
11-12	2.67 ± 0.07 [a]	2.58 ± 0.13 [a]	3.82 ± 0.18 [b]	3.81 ± 0.42 [b]
13-14	2.56 ± 0.12 [a]	2.44 ± 0.11 [a]	3.82 ± 0.06 [b]	3.31 ± 0.19 [b]
Total	248.85 ± 9.12 [a]	238.94 ± 11.22 [a]	493.87 ± 6.22 [b]	472.83 ± 24.89 [b]

Different letters indicate that there is statistical significance in comparison between groups， $P < 0.05$

Table S2. Average energy intake of mice

Weeks	Calorie intakes (kcal/d)			
	ND + water	ND + MP	HFD + water	HFD + MP
1-2	9.76 ± 0.34 [a]	9.67 ± 0.32 [a]	37.55 ± 6.87 [b]	42.14 ± 1.80 [b]
3-4	8.90 ± 0.37 [a]	8.20 ± 0.50 [a]	27.00 ± 2.28 [b]	24.69 ± 2.56 [b]
5-6	9.47 ± 0.49 [a]	8.83 ± 0.48 [a]	25.88 ± 1.92 [b]	23.11 ± 1.69 [b]
7-8	11.32 ± 0.49 [a]	11.31 ± 0.57 [a]	29.78 ± 1.06 [b]	28.29 ± 2.56 [b]
9-10	8.84 ± 0.40 [a]	8.38 ± 0.38 [a]	24.61 ± 2.85 [b]	21.46 ± 2.07 [b]
11-12	10.29 ± 0.29 [a]	9.92 ± 0.49 [a]	20.03 ± 0.93 [b]	19.95 ± 2.21 [b]
13-14	9.85 ± 0.45 [a]	9.39 ± 0.43 [a]	20.00 ± 0.29 [b]	17.33 ± 0.99 [b]
Total	958.07 ± 35.12 [a]	919.93 ± 43.19 [a]	2587.86 ± 32.57 [b]	2477.65 ± 130.42 [b]

Different letters indicate that there is statistical significance in comparison between groups, $P < 0.05$

Table S3. Absolute weight of mouse viscera and tissue.

Organ	Absolute weights (g)			
	ND + water	ND + MP	HFD + water	HFD + MP
Liver	0.84 ± 0.08 [a]	0.82 ± 0.11 [a]	1.09 ± 0.30 [a]	1.12 ± 0.47 [a]
Pancreas	0.10 ± 0.02 [a]	0.09 ± 0.02 [a]	0.14 ± 0.04 [b]	0.15 ± 0.04 [b]
Fat	0.38 ± 0.12 [a]	0.44 ± 0.10 [a]	1.89 ± 0.89 [b]	2.23 ± 0.41 [b]
Caecum	0.23 ± 0.07 [a]	0.22 ± 0.05 [a]	0.18 ± 0.04 [b]	0.20 ± 0.05 [b]

Different letters indicate that there is statistical significance in comparison between groups, $P < 0.05$

Table. S4. Relative weight of organs and tissues in mice.

Organ	Relative weights (g)			
	ND + water	ND + MP	HFD + water	HFD + MP
Liver	3.27 ± 0.14 [a]	3.13 ± 0.26 [a]	2.83 ± 0.46 [a]	2.76 ± 0.70 [a]
Pancreas	0.40 ± 0.10 [a]	0.36 ± 0.06 [a]	0.36 ± 0.12 [b]	0.37 ± 0.08 [b]
Fat	1.45 ± 0.49 [a]	1.61 ± 0.49 [a]	4.83 ± 1.95 [b]	5.73 ± 1.00 [b]
Caecum	0.89 ± 0.20 [a]	0.83 ± 0.14 [a]	0.47 ± 0.14 [b]	0.52 ± 0.15 [b]

Different letters indicate that there is statistical significance in comparison between groups, $P < 0.05$
